# Galectin-3 released in response to traumatic brain injury acts as an alarmin orchestrating brain immune response and promoting neurodegeneration

**DOI:** 10.1038/srep41689

**Published:** 2017-01-27

**Authors:** Ping Kei Yip, Alejandro Carrillo-Jimenez, Paul King, Anna Vilalta, Koji Nomura, Chi Cheng Chau, Alexander Michael Scott Egerton, Zhuo-Hao Liu, Ashray Jayaram Shetty, Jordi L. Tremoleda, Meirion Davies, Tomas Deierborg, John V. Priestley, Guy Charles Brown, Adina Teodora Michael-Titus, Jose Luis Venero, Miguel Angel Burguillos

**Affiliations:** 1 Centre for Neuroscience and Trauma. Blizard Institute. Queen Mary University of London, E1 2AT London, United Kingdom; 2 Departamento de Bioquímica y Biología Molecular, Facultad de Farmacia, Universidad de Sevilla, and Instituto de Biomedicina de Sevilla-Hospital Universitario Virgen del Rocío/CSIC/Universidad de Sevilla, 41012 Sevilla, Spain; 3 Department of Biochemistry, University of Cambridge, Cambridge CB2 1QW, United Kingdom; 4 Chang Gung Medical College and University, Chang Gung Memorial Hospital, Department of Neurosurgery, 5 Fu-Shin Street, Linkou, Taiwan; 5 Experimental Neuroinflammation Laboratory, Department of Experimental Medical Science, Lund University, BMC B11, 221 84 Lund, Sweden

## Abstract

Traumatic brain injury (TBI) is currently a major cause of morbidity and poor quality of life in Western society, with an estimate of 2.5 million people affected per year in Europe, indicating the need for advances in TBI treatment. Within the first 24 h after TBI, several inflammatory response factors become upregulated, including the lectin galectin-3. In this study, using a controlled cortical impact (CCI) model of head injury, we show a large increase in the expression of galectin-3 in microglia and also an increase in the released form of galectin-3 in the cerebrospinal fluid (CSF) 24 h after head injury. We report that galectin-3 can bind to TLR-4, and that administration of a neutralizing antibody against galectin-3 decreases the expression of IL-1β, IL-6, TNFα and NOS2 and promotes neuroprotection in the cortical and hippocampal cell populations after head injury. Long-term analysis demonstrated a significant neuroprotection in the cortical region in the galectin-3 knockout animals in response to TBI. These results suggest that following head trauma, released galectin-3 may act as an alarmin, binding, among other proteins, to TLR-4 and promoting inflammation and neuronal loss. Taking all together, galectin-3 emerges as a clinically relevant target for TBI therapy.

Traumatic brain injury (TBI) has become one of the main causes of death and disability in the Western world, where ~160,000 admissions to hospital were catalogued as head injury during the period 2013–14 in the UK (data obtained from the Headway brain injury association). TBI pathology results in a complex set of symptoms that may lead to long-term impaired cognitive function and dementia, Parkinson’s disease, and amyotrophic lateral sclerosis^1^. Prompt actions in both pre-hospital and early in-hospital stay are considered as key factors to decrease mortality and improve the patient’s neurological outcome^2^. Despite recent advances in the management of TBI, its consequences remain significant and often very disabling^3^.

TBI can be divided into several stages: (i) the primary injury (due to the initial impact, e.g. contusion of the brain); (ii) the secondary injury, characterized by the spread of cell loss, diffuse axonal damage and also a multiphasic neuroinflammatory response and (iii) the stage characterized by attempted, but ineffective, regeneration and repair. Many of the current efforts to reduce the damage after head injury are focused on the events that occur during the secondary injury phase and in particular those regulating the neuroinflammatory response^4^. Although it is well established that the neuroinflammatory process plays an important role during TBI, it is still not clear how to modulate it in a manner that provides beneficial results^4^,^5^. Recent studies that have focused on the inflammatory response after head injury, demonstrate that microglia/macrophages display a mixed phenotype as a result of the complex signalling environment instead of a well-defined “M1” or “M2” phenotype^6^,^7^,^8^. Eventually, the M2-like phenotype turns into a more proinflammatory phenotype which increases the cortical and hippocampal neurodegeneration^8^.

Various therapies to treat TBI have been shown in experimental animal models to be effective when they are dosed before or soon after TBI^9^. This highlights the importance of the early events triggered after head injury, for TBI progression. One of the first defence mechanisms that is activated after head injury is the innate immune response. Activation of members of the Toll-like Receptor (TLR) family has been shown to play an important role in different CNS injuries or infections and during the progression of different neurodegenerative diseases^10^. The release of alarmins or damage-associated molecular pattern molecules (DAMPs) upon injury, activates pattern recognition receptors (PRRs), such as TLRs^11^. Among the different TLR members, TLR-2 and TLR-4 have been shown to play a key role during the neuroinflammatory response in various experimental models for TBI^12^,^13^,^14^,^15^,^16^,^17^,^18^. Therefore, finding treatments to decrease TLR-2 and TLR-4 activation would be therapeutically advantageous.

Galectins are a family of proteins that consist of 15 members with significant sequence similarity in their carbohydrate-recognition domain (CRD) with affinity towards β-galactosidases^19^. Galectin-3 has been shown to have different functions depending on cell type and cellular location. Galectin-3 can be found inside the nucleus and the cytosol, embedded in the plasma membrane^20^ or released extracellularly upon exposure to certain stimuli such as lipopolysaccharide (LPS)^21^,^22^ or IFNγ^23^. Galectin-3 appears to function as a master regulator during the inflammatory response in neurodegenerative diseases. We have recently demonstrated that galectin-3 is released by activated microglia in response to proinflammatory stimuli and importantly, galectin-3 acts as an endogenous paracrine TLR4 ligand^22^. We also demonstrated that released galectin-3 is essential for the full microglial response upon LPS treatment, thus supporting a central role of this galectin in regulating brain immune response. Furthermore our previous studies showed that: (i) galectin-3 is involved in the proinflammatory response triggered by α-synuclein in microglial cells^24^, a hallmark of Parkinson’s disease physiopathology, and (ii) mice lacking galectin-3 were more resistant to hippocampal degeneration in a model of global cerebral ischemia that mimics the brain damage caused by cardiac arrest^22^. Regarding the role of galectin-3 under conditions of brain trauma, several studies have demonstrated striking early increases in the expression of galectin-3 in different trauma models including spinal cord injury^25^,^26^,^27^ and also in experimental models of TBI^28^,^29^. Interestingly, a recent study performed in 8 weeks post-contusion spinal cord injured mice, found galectin-3 as one of the most upregulated extracellular proteins^30^, suggesting that galectin-3 plays also a role at later stages of the injury. Consequently, and given the important role of galectin-3 in regulating brain inflammation and neurodegeneration, studies aimed to elucidate the role of this galectin after TBI are encouraged. In this study, we focus on the role that galectin-3 plays during the early neuroinflammatory response driven by microglia cells after head injury.

## Results

### Galectin-3 is expressed mainly in microglia cells and released after cortical impact

**Figure 1:**
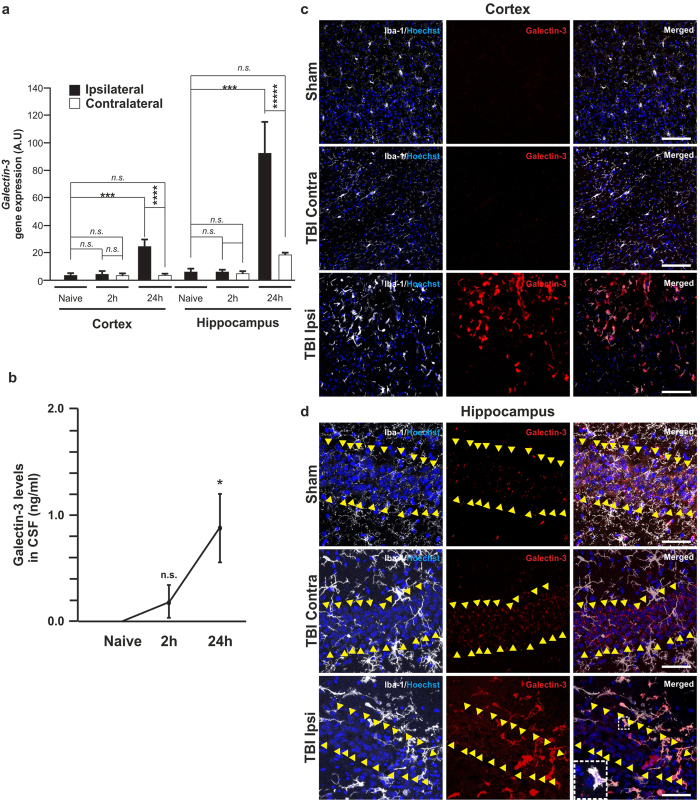
Galectin-3 is highly expressed and released in the brain after TBI in a cortical contusion murine model. qRT-PCR analysis of galectin-3 expression at 2 and 24 h after TBI in the cortex and hippocampus (**a**). CSF protein levels of galectin-3 at 2 and 24 hours after TBI (**b**). Images showing colocalization of galectin-3 positive cells with Iba-1 in sham, TBI contralateral and TBI ipsilateral in Cortex (**c**) and Hippocampus (**d**) 24 h after TBI. Statistics and error bars: mean ± s.d. n = 4 for A and n = 5 for B. Data were analyzed using a two-sided Student’s t-test. * p < 0.05, ***p < 0.001 and ****p < 0.0001. Scales bar for c is 100 μm and for d is 50 μm. Arrowheads define the area of neuronal cell bodies in the CA1 region. Hoechst was used to counterstain the nuclei.

We used a cortical contusion mouse model to investigate when and where galectin-3 is expressed after head injury. Total RNA was extracted from cortex and hippocampus to quantify galectin-3 levels (Fig. 1a). We found a clear induction of galectin-3 expression 24 h after impact on the ipsilateral site in both cortex and hippocampus, and the levels remained high thereafter, even 3 weeks later (data not shown). Although induction of galectin-3 could not be observed 2 h after head injury using qPCR, we found some high galectin-3 expressing cells by immunohistochemistry in both cortex and hippocampus (Supplementary Fig. S1). We also investigated whether galectin-3 is released into brain parenchyma after head injury. Consequently, we analysed the content of galectin-3 in cerebrospinal fluid (CSF) after head injury as compared to naïve animals and we observed a statistically significant increase in galectin-3 in the CSF 24 h after the impact (Fig. 1b). We then decided to study which cells were predominantly expressing galectin-3 24 h after the injury through an immunohistochemical colocalization analysis. We observed that the majority of cells highly expressing galectin-3 were Iba-1 positive cells (Fig. 1b and d). To confirm more specifically whether those Iba-1 positive cells expressing galectin-3 were microglia, we used the specific microglia marker TMEM119^31^. This analysis confirmed that microglia express galectin-3 under conditions of brain trauma (Supplementary Fig. S1). Astrocytes have been shown to be able to express high levels of galectin-3 five days post injury (dpi) in a stab wound-injured cerebral cortex^32^. However, the number of GFAP-labelled astrocytes expressing galectin-3 24 h after the contusion was very low (Supplementary Fig. S1). Interestingly, we could find a milder punctiform galectin-3 staining present in cells belonging to the pyramidal cell layer in the CA1 region on both the ipsilateral and contralateral sites 24 h after injury (Fig. 1d). We also observed punctiform galectin-3 containing cells in NeuN positive cells in the cortex (Supplementary Fig. S1). Neuronal expression of galectin-3 has already been described in cultures of mouse dorsal root ganglion (DRG) neurons^33^ and also in the developing and adult CNS in a subset of DRG neurons in the spinal ganglia^34^.

The hippocampal formation, which is organized into very well defined neuronal layers, is highly affected by head trauma, thus allowing the study of interactions between reactive microglia and neurodegenerative processes. Iba-1 immunohistochemistry clearly demonstrated the presence of reactive microglia (based on morphological features) in the ipsilateral and contralateral hippocampus (Fig. 1d), especially when compared to sham (craniotomy) controls. Strikingly, the expression of galectin-3 was restricted to the ipsilateral cortex and hippocampus (compare TBI ipsilateral side *versus* contralateral side in Fig. 1c and d). Most galectin-3 expressing cells were iba-1 positive (Fig. 1c and d). Interestingly, these galectin-3 positive cells were consistently present in the degenerating ipsilateral hippocampal neuronal cell layers (see the high number of expressing cells invading the ipsilateral CA1 area in Fig. 1d compared with the contralateral side).

Interestingly, some of the Iba-1 positive cells were apparently surrounding other cells within the CA1 region (see detail in Fig. 1d). Microglia can remove dead, dying and stressed neurons in a process termed phagoptosis^35^. Since galectin-3 is released after trauma, and the phagocytic microglial receptor MerTK has been shown to bind galectin-3 acting as an opsonin^35^ we wondered if exogenous galectin-3 participates in the phagocytic response driven by microglia cells. To test our hypothesis, we pre-treated a murine microglial cell line (BV2) with recombinant galectin-3 for 18 h to assess the effect on the phagocytic response towards neuronal debris. We observed a remarkable 4-fold increase in the phagocytic capacity of BV2 cells in those cells pre-treated with galectin-3 compared to control (Supplementary Fig. S1). We decided then to analyse the capacity of recombinant galectin-3 to induce neuronal loss in rat primary mixed glial cultures (85% neurons). We observed a decrease in neuronal survival after 96 h treatment with recombinant galectin-3 (Supplementary Fig. S1). These results demonstrate the involvement of galectin-3 in the microglial phagocytic response and support its potential role in primary phagocytosis or phagoptosis^35^.

**Figure 2:**
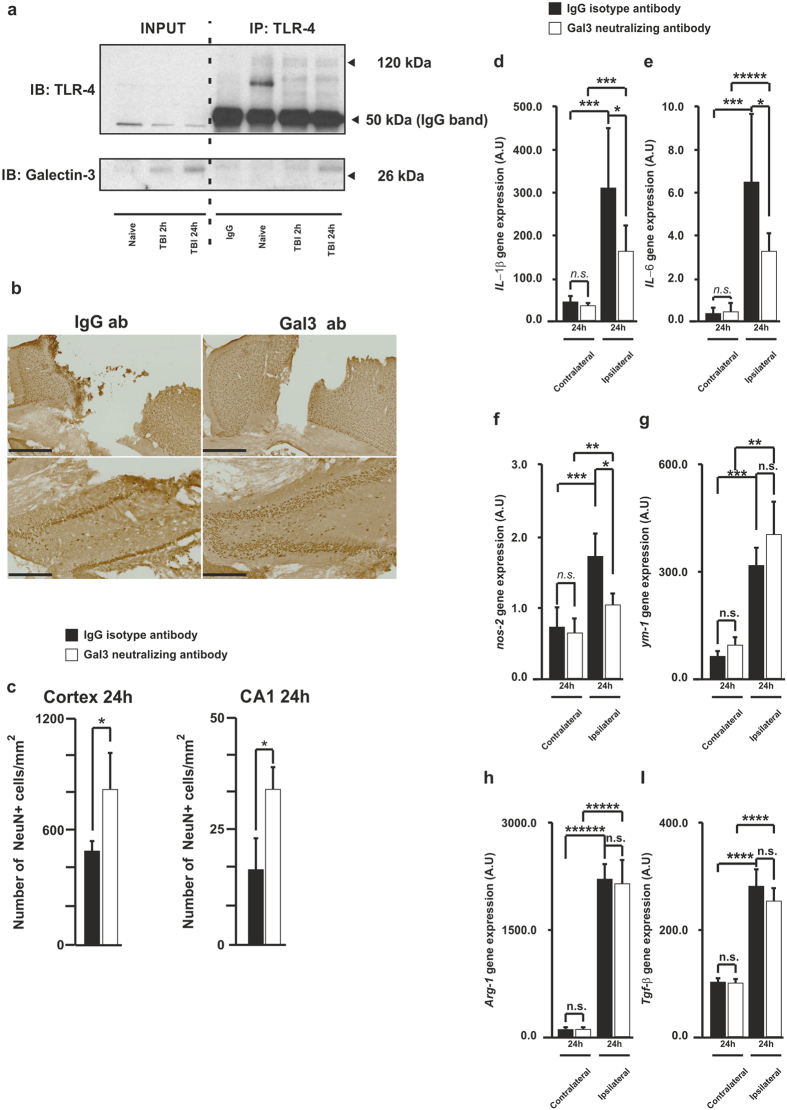
Effect on the inflammatory response and neuronal survival after TBI in mice injected with neutralising antibody against galectin-3 compared to isotype IgG control. Analysis of the binding of TLR-4 and galectin-3 in the whole tissue extract at different time points after immunoprecipitating with TLR-4 antibody (**a**) followed by incubation with TLR-4 antibody (**a**, upper panel) or with galectin-3 antibody (**a**, lower panel). Immunohistochemistry of NeuN (**b**) and quantification (**c**) in the cortex and hippocampus of mice injected with neutralising antibody against galectin-3 or isotype control. Quantification of the gene expression in whole tissue for IL-1β (**d**), IL-6 (**e**), NOS-2 (**f**), Ym-1 (**g**), Arg-1 (**h**) and TGF-β (**i**). Statistics and error bars: mean ± s.d. n = 4 for c, n = 7 for (**d–i**). Data were analysed using two-sided Student’s t-test. *p < 0.05. Scale bar in b is 250 μm.

Recently, our group has demonstrated that the released form of galectin-3 may act as a TLR4 agonist inducing a proinflammatory response under ischemic conditions^22^. We wanted to know whether the released form of galectin-3 after contusion might play also a role as a TLR-4 ligand^22^. For this reason, we analysed the interaction between TLR4 and galectin-3 through an immunoprecipitation approach. Protein extracts obtained from naïve, 2 h and 24 h CCI-injured mice were immunoprecipitated overnight with a TLR-4 antibody. The immunoprecipitate was collected and run on a gel, and then transferred into a nitrocellulose membrane. After that, the membrane was immunoblotted with a specific galectin-3 antibody. The results showed an interaction between galectin-3 and TLR4 at 24 h but not at 2 h after injury (Fig. 2a, cropped blots, full-length blot is presented in Supplementary Fig. S2) in correspondence with the TBI-induced released levels of galectin-3 as measured in the CSF (Fig. 1b).

### Galectin-3 neutralising antibodies reduce inflammation, microglial activation and neuronal loss after head injury

To further study the role of the released form of galectin-3 during the inflammatory response, we injected into the contused cortex 0.5 μg of either neutralizing antibody against galectin-3 or control isotype antibody (total volume injected was 5 μl at a rate of 1 μl/min) 1 hour after the contusion. We assessed the effect of the neutralizing antibody on neuronal survival in the cortex and hippocampus and the inflammatory response 24 h after injury. Interestingly, we observed a neuroprotective effect of galectin-3 neutralizing antibody in the cortex and hippocampus 24 h after head injury (Fig. 2b,c). TBI triggers a very potent neuroinflammatory response which is responsible for many of the deleterious effects during secondary injury^36^. Microglia and macrophages express both M1 and M2 markers early after injury, but with time, the transient upregulation of M2 markers is replaced by a more M1-like phenotype^8^. Consequently, we analysed the mRNA levels of key M1 and M2 markers in response to neutralizing or control isotype antibodies 24 h after brain trauma. Treatment with galectin-3 neutralizing antibody reduced the trauma-induced increases of the different proinflammatory markers IL-1β, IL-6 and NOS2 (Fig. 2d–f). In contrast, neutralizing antibodies failed to alter the trauma-induced mRNA expression of the M2 markers Ym-1, Arg-1 and TGF-β (Fig. 2g–i). It is well known that microglia activation is accompanied by graded morphological alterations; thus early-stage activation is characterized by increased ramification of cytoplasmic processes, cell size and enhanced Iba-1 expression. After that, it is followed by further thickening of processes and retraction of the finer ones and increased cell body size to end up with a complete retraction of cytoplasmic processes, thus acquiring amoeboid-shape morphology. We analysed the effect that the injection of the galectin-3 neutralizing antibody had on different microglia morphology features 24 h after head injury. The results showed that microglia in mice injected with the neutralizing antibody exhibited a less “amoeboid” morphology with higher numbers of secondary branches and triple and quadruple junction points as compared to microglia from animals treated with IgG isotype control antibody (Supplementary Fig. S3).

### Genetic knockout of galectin-3 reduces neuronal loss after head injury and changes microglial morphology

**Figure 3:**
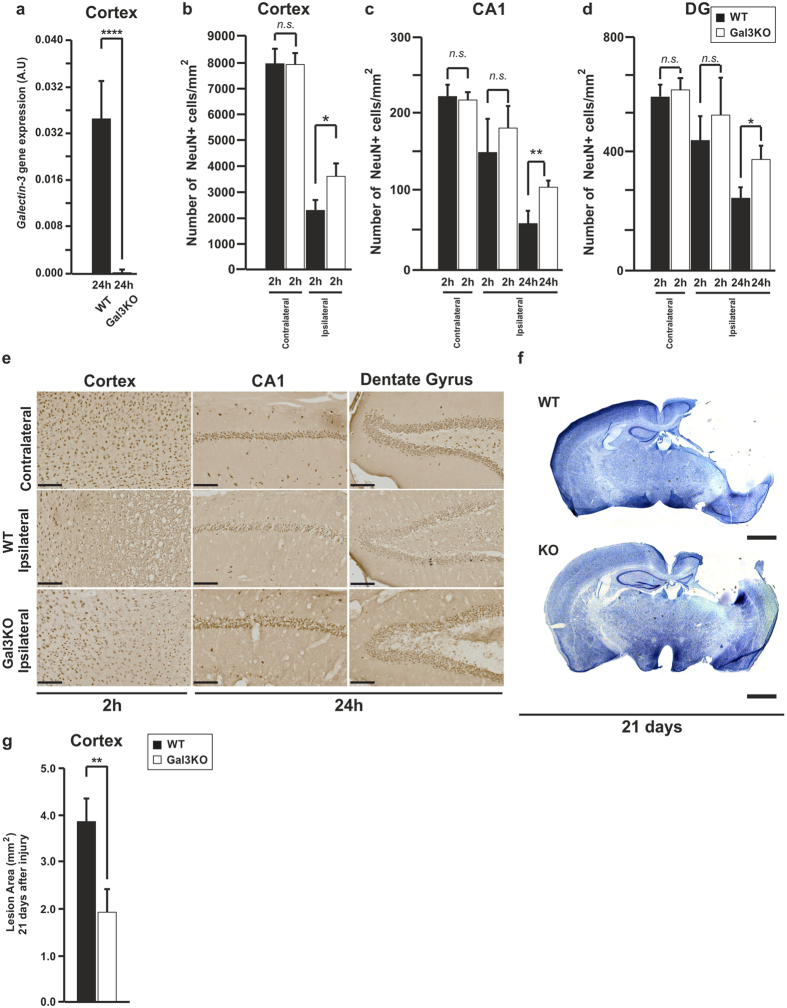
Effect of the lack of galectin-3 on the neuronal survival in cortex and hippocampus after TBI. Chart comparing the expression of galectin-3 between wild type and galectin-3 knockout mice in the ipsilateral cortex 24 h after injury (**a**). Comparison of NeuN-positive cells between wild type and galectin-3 knockout mice after TBI in the cortex (**b**), CA1 (**c**) and dentate gyrus (**d**). Representative pictures of NeuN in different regions (**e**). Representative image of toluidine blue staining in wild type and galectin-3 knockout mice (**f**) and quantification of the cortical tissue loss (**g**) 21 days after injury. Statistics and error bars: mean ± s.d. n = 4 for (**a–d**) and (**g**). Data were analysed using two-sided student’s t-test. *p < 0.05 **p < 0.01 and ****p < 0.0001. Scale bar in e is 100 μm and for f is 1000 μm.

Galectin-3 can play diverse roles in the regulation of different processes such as proliferation, adhesion, migration, invasion, angiogenesis, apoptosis and inflammation^37^,^38^,^39^. It is noteworthy that in some of these processes opposite effects have been reported (as for instance in the control of the inflammatory response)^23^,^40^, due to a different location within the cell and/or posttranslational modification. We wanted to analyse the effect that depletion of galectin-3 could have over the neuronal population after head injury using galectin-3 knockout mice (Fig. 3a). We first decided to analyse the survival of the neuronal population by analysing the number of NeuN-positive cells in three different regions (Cortex, CA1 pyramidal cell layer and Dentate Gyrus -DG-) within the first 24 h after injury. The results showed that lack of galectin-3 is neuroprotective within the first 2 h in the cortical region (Fig. 3b), while in the case of the hippocampal area we needed to wait until 24 h to observe a neuroprotective effect in the CA1 and DG regions (Fig. 3c,d). Figure 3e shows representative images of the three different regions (cortex, CA1 pyramidal layer and DG) in wild type and galectin-3 knockout mice within 24 h after contusion. We wanted to know if this neuroprotective effect within the first 24 h was long-lasting. For that reason, we analysed using toluidine blue staining the area of loss in the cortex 21 days after injury (Fig. 3f,g) in wild type and galectin-3 knockout mice. We found that lack of galectin-3 protected against the long-term TBI-induced cortical damage, confirming the observed neuroprotective effect at earlier time points.

**Figure 4:**
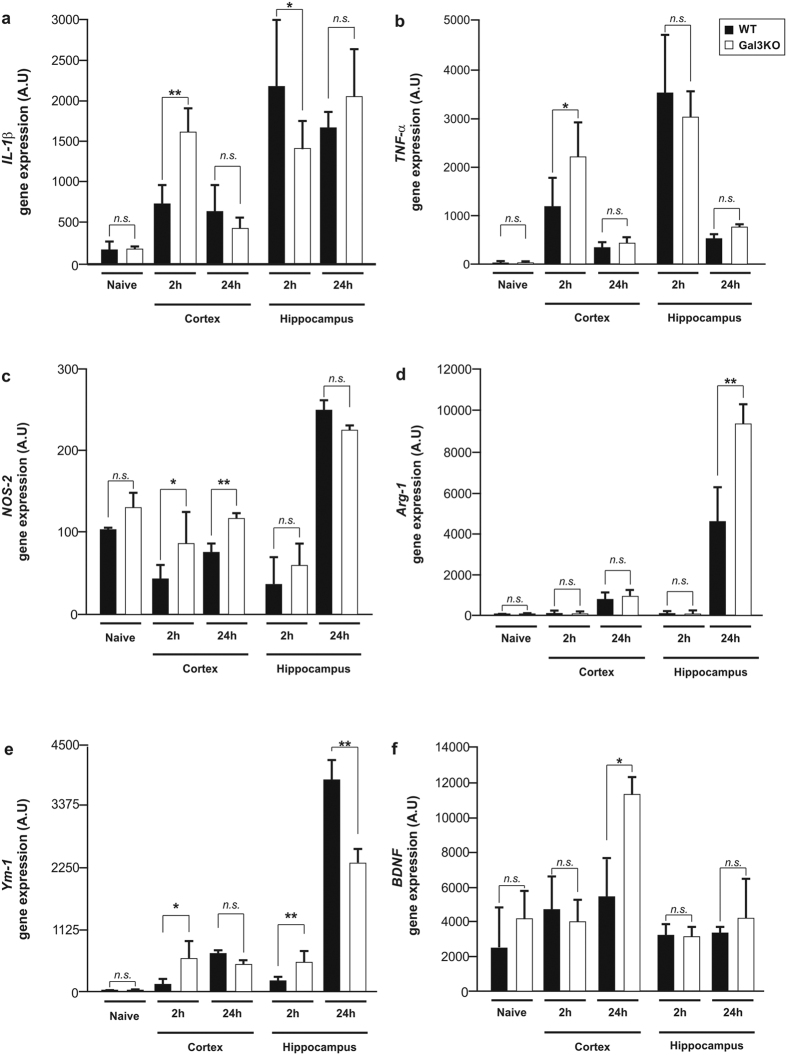
Assessment of the inflammatory response in wild type and galectin-3 knockout mice after TBI. Analysis of the gene expression on the ipsilateral side of the isolated cortex and hippocampus in wild type and galectin-3 knockout mice for IL-1β (**a**), TNF-α (**b**) and NOS-2 (**c**), Arginase-1 (**d**), Ym1 (**e**) and BDNF (**f**). Statistics and error bars: mean ± s.d. n = 5. Data were analysed using a two-sided Student’s t-test followed by one-way ANOVA with post-hoc Fisher’s least significant difference (LSD). *p < 0.05 **p < 0.01.

We analyzed the neuroinflammatory response through the expression of several factors responsible for inducing either a proinflammatory or a supportive environment (Fig. 4) in the cortex and hippocampus early after impact. Most remarkable effects were found in the M2 markers analysed. Specifically, galectin-3 deficiency further increased the trauma-induced mRNA up-regulation for arginase-1 (Arg-1) in the hippocampus at 24 h and of Ym-1 in the cortex at 2 h (Fig. 4d,e). We also analysed two key neurotrophins, BDNF and NGF. Interestingly, galectin-3 knockout animals exhibited significantly higher cortical mRNA levels of BDNF as compared with wild type animals after trauma (Fig. 4f), while NGF remained unaltered (Supplementary Fig. S4). Regarding the pro-inflammatory response, TBI did up-regulate all markers analyzed, including IL-1β, TNF-α and NOS-2 in the cortex and hippocampus in WT animals (Fig. 4a–c). Unexpectedly, galectin-3 deficiency exacerbated transiently the trauma-induced upregulation of all M1 markers in cortex 2 h after injury (Fig. 4a–c). This highlighted a discrepancy in the immune response to TBI between galectin-3 deficient mice and mice injected with anti-galectin-3 neutralising antibody. This may be explained by the difference between a total constitutive genetic deficiency of galectin-3, affecting all cell types and compartments, versus a model using a neutralising antibody against galectin-3, affecting only extracellular galectin-3.

Rod microglia have been shown to play a role during the proinflammatory response in two different experimental models of head trauma^41^,^42^. Consequently, we decided to analyse the number of rod microglia early after head trauma. We focused on two areas where the appearance of the rod microglial cells (CA1 and the somatosensory cortex) has been described after head trauma. We observed a decrease in the presence of rod microglia in the galectin-3 knockout mice (Supplementary Fig. S4), and of microglia positioned along the axons of neurons (Supplementary Fig. S4).

## Discussion

One of the major challenges in TBI research is to identify new therapies or molecules that improve the patient’s neurological outcome after injury. The secondary injury occurring after TBI contributes to a worsening of the neurological outcome in addition to the initial contusion. Identifying factors that regulate the TBI-induced neuroinflammatory response could generate candidates for therapies.

Soon after a head or spinal cord injury, the expression of several proteins related to the inflammatory response is upregulated, including the lectin galectin-3^25^,^26^,^27^,^28^,^29^. The neuroinflammatory response triggered by TBI is believed to be a major determinant of the secondary cell death after brain trauma. We have previously demonstrated that reactive microglia release galectin-3, thus playing a major role in the microglial immune response during ischemia^22^. Our goal in this study was to investigate the role of galectin-3 during head injury, and whether there was any release of galectin-3 after TBI. The idea that there could be an increase in the released form of galectin-3 after head injury was suggested by different studies demonstrating an increase in the levels of galectin-3 in plasma after TBI^43^,^44^. Interestingly in one of these studies, the authors found a positive correlation between the galectin-3 levels in human plasma and the Glasgow Coma Scale (GCS) scores^43^. In our study, we found significant amounts of galectin-3 in the mouse CSF 24 h after injury. The blood brain barrier is highly disrupted immediately after head injury^45^, therefore a substantial leakage of galectin-3 from the brain parenchyma into the CSF can be expected.

Our next goal was to identify which cell types express galectin-3. Confocal immunofluorescence using antibodies to TMEM119 (specific to microglia^31^), Iba-1 (microglia and macrophages), GFAP (astrocytes) and NeuN (neurons) along with galectin-3 antibodies led us to confirm that: (i) Iba-1 positive cells are the main cells expressing galectin-3, (ii) reactive microglia, as demonstrated by TMEM119 expression, have also high levels of galectin-3, (iii) the majority of astrocytes lack galectin-3 expression within the first 24 h after impact, and (iv) most neurons do not express galectin-3, but unexpectedly certain neuronal cell types cells do express galectin-3.

We investigated the role(s) that extracellular galectin-3 may play during TBI. We have recently described a new role for released galectin-3 as an endogenous ligand for TLR-4, triggering a TLR-4-dependant neuroinflammatory response^22^. In our TBI model we observed that galectin-3 binds to TLR-4. Importantly, TLR-4 activation has been shown to modulate the early neuroinflammatory response after head injury, and inhibition of TLR-4 promotes a better neurological outcome^15^,^16^,^17^. By using a neutralizing antibody against galectin-3 we could observe a decrease in the expression of IL-1β, IL-6 and NOS2 but no difference in any of the M2 markers upon head injury.

There are studies where galectin-3 has been described to act as opsonin/bridging molecule by simultaneously binding to Mer receptor tyrosine kinase (MerTK) and “eat-me” signals on neurons thereby stimulating phagocytosis^35^,^46^,^47^. Hence, one or more roles for the extracellular galectin-3 could occur at the same time in our model. The possibility that released galectin-3 may have an important role in modulating phagocytosis is certainly attractive. Consistent with this view we observed the presence of highly expressing galectin-3 reactive microglia/macrophages close to hippocampal cells and restricted to the pyramidal cell layer, which is highly sensitive to brain trauma. To ascertain a potential role of galectin-3 in promoting phagocytosis, we conducted *in vitro* culture assays using the microglial cell line BV2 exposed to TAMRA-labelled neuronal debris and pre-treated with exogenous galectin-3. FACS analysis demonstrated a positive role of exogenous galectin-3 in promoting microglial phagocytosis of neuronal debris. We also found that exogenous galectin-3 increased cell death in primary mixed glial cultures. Since phagocytosis can be extended to viable neurons, a process known as primary phagocytosis or phagoptosis, the possibility that galectin-3 participates in this process is plausible. Our results suggest an involvement of galectin-3 during the phagocytic response after TBI, but still more in depth studies are required to confirm this hypothesis.

Once we established a potential role of galectin-3 in promoting phagocytosis and cell death, we next analysed the potential effect of galectin-3 gene deletion in mice subjected to contusion TBI. A significant protection of cortical and hippocampal neurons was found in animals lacking galectin-3 at early and late time points (21 days) supporting our hypothesis that galectin-3 plays a deleterious role early after head trauma conditions.

We and others have studied the immunomodulatory properties of galectin-3 towards the two opposite polarization states of microglia or macrophages^21^,^22^,^23^,^24^. TBI induces a complex environment where a mixed inflammatory response (M1 and M2 phenotypes) develops early after injury^6^,^7^,^8^. We observed a similar pattern in our own analysis of the inflammatory response. Consequently, we analysed the effect of galectin-3 gene deletion on different markers of microglia polarization including IL-1β, TNF-α and NOS-2 (M1) and arginase-1 and Ym-1 (M2) in response to TBI. Unexpectedly, galectin-3 gene deletion failed to modify the trauma-induced proinflammatory response, thus contrasting with the anti-inflammatory effect of galectin-3 neutralizing antibodies. However, it should be stressed that the hippocampus exhibited a dramatic increase in classical M2 activation markers 24 h after trauma (i.e. arginase-1). The reason for this discrepancy may be a consequence of the plethora of functions of galectin-3 depending on where it is located within the cell (released, cytosolic or nuclear) and the cell type in which it is expressed^20^,^21^,^22^,^23^,^32^. The disparity observed in terms of M1 markers in response to either galectin-3 deletion (intra and extracellular) or neutralizing antibodies (extracellular) highlights the complex galectin-3-dependent cellular interactions that regulate brain immune functions. Galectin-3 gene deletion decreased the number of “rod microglia” shortly after brain trauma, thus supporting a role of galectin-3 in the proinflammatory response to brain trauma. Our study demonstrates that galectin-3 could be working as an endogenous ligand for TLR4 24 h after brain trauma, at a time post-injury when galectin-3 mRNA and protein levels are high. These results suggest that galectin-3 might be, together with other DAMPs, responsible for the full activation of different immune cells via TLR activation within the first 24 h. Therapies aimed at modulating galectin-3 expression or availability within the first 24 h after injury might have a potentially positive effect on patients’ outcome at later time points (as was observed in the galectin-3 knockout mice 21 days after injury).

Overall, we conclude that galectin-3 acts as an alarmin under conditions of brain trauma, and induces a strong proinflammatory response, most likely through TLR4 activation, and might be involved also in the phagocytic response. Considering that the facilitation of M2 activation may be a promising strategy to promote tissue repair under conditions of brain trauma, pharmacological inhibition of galectin-3 deserves special attention. In support of this view, galectin-3 gene deletion further exacerbated the trauma-induced M2 response in the hippocampus. Aside from exhibiting enhanced phagocytosis, the M2 phenotype is associated with the release of trophic factors. We decided to analyse the effect of brain trauma and galectin-3 gene deletion on the mRNA levels of NGF and BDNF. We did not find any differences in the levels of NGF but strikingly, the lack of galectin-3 markedly increased the cortical mRNA levels of BDNF in response to brain trauma, with no response in the hippocampus. BDNF can be synthetized not only in neurons but also in astrocytes^48^ and microglia^49^. We could not observe changes in BDNF expression in mice treated with neutralizing antibody against galectin-3 (data not shown), suggesting that only intracellular galectin-3 is responsible for changes in BDNF expression. At the time point at which BDNF was upregulated (24 h), the majority of cells expressing galectin-3 were either neurons or microglia but not astrocytes. This suggests that the source of BDNF is either neuronal and/or microglial but not astrocytic at the time examined.

BDNF is strongly up-regulated shortly after TBI in the hippocampus and cortex^50^,^51^ which supports a trophic role of this neurotrophin after neurotrauma conditions. Consistent with this view, several studies have demonstrated a beneficial effect of BDNF administration in different spinal cord injury models^52^,^53^,^54^. Furthermore, small molecule BDNF mimetics which specifically bind and activate trkB receptors, have been shown to improve motor learning after TBI^54^ and have neuroprotective effects in adult^55^,^56^ and newborn^57^ mice after CCI. Our results suggest an inhibitory role of galectin-3 on BDNF expression after head injury and add new evidence supporting the potential of anti-galectin-3 therapy in TBI.

In conclusion, our study demonstrates that galectin-3 is expressed and released predominantly by microglial cells in response to TBI, thus acting as an alarmin. The detrimental role of galectin-3 early after TBI was corroborated using galectin-3 knock-out mice and neutralizing antibodies, which conferred neuroprotection in response to TBI. Once released, galectin-3 binds to and activates TLR4, thus inducing a proinflammatory response and perhaps also enhancing the phagocytic response. Our data also suggest an important role of galectin-3 in regulating the M2 polarization state and BDNF mRNA expression after TBI.

## Methods

### Animals

All experiments conducted in Seville were approved by The University of Seville Ethical Committee for Experimental Research and fulfilled the requirements for experimental animal research in accordance with the Guidelines of the European Union Council (86/609/EU) and the Spanish regulations (BOE 34/11370–421, 2013) for the use of laboratory animals. All the experiments performed in London were approved by the Queen Mary University of London Animal Welfare and Ethical Review Body and carried out under Licence from the UK Home Office in accordance with the United Kingdom Animals (Scientific Procedures) Act 1986 for the use of animals in research and Directive 2010/63/EU. All experiments performed in Cambridge were performed in accordance with the UK Animals (Scientific Procedures) Act (1986) and were approved by the Cambridge University local ethical committee.

10–12 week-old not littermate wild type and galectin-3 null mutant mice on the C57BL/6 background^58^ were obtained from Dr. T. Deierborg from Lund University. 10–12 week old CD-1 (IRC) outbred mice were purchased from Charles River Laboratories. Animals were housed under a 12 h light/dark cycle with free access to food and water.

### Genotyping

The genotype of gal3−/− and gal3+/+ mice was determined by analysis of DNA extracted from the tails using a QuickExtract^TM^ (Epicentre) and amplified with the MyTaq^TM^ Red DNA Polymerase (Bioline). The PCR consisted of 94 °C for 1 min, then 35 cycles with denaturation at 95 °C for 15 sec, annealing at 58 °C for 15 sec, and elongation at 72 °C for 10 sec.

The primer sequences used were obtained from a previous study^59^: galectin-3 common 5′-CAC GAA CGT TTG CTC TCT GG-3′; galectin-3−/− 5′-GCT TTT CTG GAT TCA TCG ACT GTG G-3′ (product size on a gel: 384 bp) and galectin-3+/+ 5′-TGA AAT ACT TAC CGA AAA GCT GTC TGC-3′ (product size on a gel: 300 bp).

### Surgery

Mice were anaesthetized with a mixture of ketamine (50 mg/kg) and medetomidine (10 mg/kg) intraperitoneally (i.p.) and given pre-emptively analgesia with buprenorphine (0.05 mg/kg) subcutaneously (s.c.). Once surgical anaesthesia was confirmed, mice were placed into a stereotaxic frame and secured with teeth bar and guinea pig ear bars. A midline incision was made to expose the skull and a 3.0 mm craniotomy was made over the right parietal bone (central point was at −2.0 mm bregma, 2.5 mm lateral) using a 0.5 mm diameter burr bit connected to a hand held dental drill. Care was ensured so that the dura mater was undisturbed during the drilling and the removal of the skull flap.

The contusion was carried out using the Hatteras PinPoint controlled cortical impactor (Hatteras Instruments, USA). Parameters used were: 3.0 m/s velocity, 100 ms dwell time, and 2.2 mm impact depth at 20° angle. Sham injury mice received a craniotomy only. After the injury, the skull bone flap was replaced, skin sutured and placed into a warm incubator (27–28 °C) to recover.

In another study, agents were administered intracerebrally at 1 h post injury (final volume 5 μl of either galectin-3 neutralising antibody or IgG isotype control during 5 minutes at a rate of 1 μl/min; the concentration of the antibodies was 0.1 μg/μl). After the TBI surgery, the animal was transferred to a post-operative chamber and kept warm during the 1 h period prior to the injection into the brain. Ten minutes prior to the intracerebral injection, a top up of anaesthesia was provided to ensure the mice were fully surgically anaesthetized. The mice were secured into a stereotaxic frame and once the injury site was revealed agents were injected similarly to previously described^60^. Briefly, using a glass micropipette, 5 μl of agent was injected directly into the injury epicentre at a speed of 1 μl/min and depth of 1.5 mm, avoiding the hippocampus due to the swelling of the cortex. After injection, the glass micropipette was slowly withdrawn, the skull bone flap gently placed over the site, and the skin was sutured. Approximately 40 min after the last dose of the anaesthetic drug, antipamezole (Antisedan, 2 mg/kg, s.c.) and sterile saline (100 ml/kg, s.c.) was administered to reverse the anaesthetic effect and prevent dehydration, respectively. Post-operative care involved daily weight monitoring and twice daily administration of buprenorphine (0.05 mg/kg, s.c.) until there was weight gain or when culled at a defined time point.

At different time-points after the surgery, mice were put under deep anaesthesia before transcardially perfused with 4% paraformaldehyde solution for immunohistochemistry analysis. Mice whose brains were used to extract RNA or proteins extracts were transcardially perfused with PBS and the brains were quickly dissected and fast frozen.

### Cell culture

Murine microglial BV2 cell line (passage <30) was cultured as previously described^22^. Primary mixed neuronal/glial cultures were prepared from cerebella of postnatal day 5 to 7 Wistar rats (male and female). Neurons were plated at a density of 5 × 10^5^ cells per well in a 24-well plate and medium was changed once 24 hours after the cells were seeded. The cell cultures were left for one week before treatment. The approximate composition of the mixed cultures is 85 ± 5% neurons, 7 ± 3% astrocytes, and 5 ± 3% microglia. For live-cell counts, cultures were incubated with the nuclear stains Hoechst 33342 (5 μg/ml) and propidium iodide (1 μg/ml); Alexa 488-tagged isolectin-B4 (1 μg/ml) was used to distinguish microglia. Healthy and apoptotic (chromatin-condensed) neurons were recognized by their distinct nuclear morphology, whereas propidium iodide-positive cells (indicating membrane permeabilization) were identified as necrotic. Cell densities were evaluated using a Leica DMI6000 CS microscope. Four microscopy fields per well were quantified (around 150–200 neurons per field in control conditions), and experiments were performed with three independent culture preparations.

### Phagocytosis assay

Phagocytosis assays were performed as previously described^61^. Dead neurons and neuronal debris were obtained by scraping cells from live neuronal-glial culture (85% neurons), and then passing cells 10 times through a 0.4 × 13 mm needle. The resulting dead neurons and debris was stained with the succinimidyl ester of 5-carboxytetramethylrhodamine (TAMRA), and 30 μg of protein was added to BV2 microglia for 2 hours. Microglia were then washed, and the uptake of dead neurons and debris into microglia quantified by flow cytometer (Accuri C6 flow cytometer, BD) as the mean fluorescence in the red channel (FL3) per cell (10,000 cells analysed per well). Where indicated, BV-2 cells were treated ±200 nM galectin-3 (Sigma catalogue number G-5170) for 18 hours prior to addition of neuronal debris into the culture medium.

### Immunohistochemistry

Coronal sections between Bregma +0.50 to −4.04 were cut at 20 μm using a cryostat which contained the impact site and were thaw mounted onto gelatinised Superfrost Plus slides. Sections that correspond to injury epicentre and penumbra were subjected to antigen retrieval with antigen unmasking solution (H-3300, Vector Labs) at 95–100 °C for 20 min followed by another 20 min at room temperature. For those sections developed using the chromogen 3,3′ diaminobenzidine (DAB), there was an extra incubation step afterwards for 15 min in a solution of 3% H_2_O_2_ diluted in 100% methanol to quench the endogenous peroxidase activity. Thereafter, the sections were blocked at room temperature in PBS-0.1% Triton X-100 with 10% normal donkey serum (Jackson Labs, UK). Later on, the slides were incubated with the indicated primary antibodies specified in the Supplementary Table S1. Slides were washed 3 times with PBS, and then incubated with the appropriate secondary antibodies (Supplementary Table S2) followed by one-hour room temperature incubation with the Vectastain ABC kit (Vector laboratories, catalogue number PK-4000) for the immunohistochemistry developed with DAB. The peroxidase reaction was visualized using the DAB Peroxidase (HRP) Substrate Kit (with Nickel), 3,3′-diaminobenzidine (Vectashield) reaction for 2–5 min. For DAB immunohistochemistry we employed a NanoZoomer-XR Digital slide scanner (Hamamatsu, Japan).

For fluorescent immunostaining, the staining process of slides was similar to DAB staining, but fluorescent secondary antibodies were used (Supplementary Table S2). Hoechst was used to counterstain the nuclei. For the visualization and analysis of the immunofluorescence we used a Zeiss upright 710 confocal microscope with a Meta spectral head and a Leica epifluorescence microscope.

### Immunohistochemistry data analysis

Cortical and hippocampal measurements of the cell death and neuroinflammatory response following cortical impact were performed on 3 different regions (0.263 mm^2^ each region) in the ipsilateral cortex and in the different hippocampal subregions Cornu Ammonis 1 (CA1), and dentate gyrus (DG). Viable pyramidal neurons were identified as NeuN-positive cells with typical round homogenous immunoreactivity; the microglial distribution was quantified using Iba-1 staining. The quantitative analysis was carried out by performing a manual assessment of viable neurons using a 20x objective. The final numbers were obtained by multiplying the number of NeuN positive cells counted by the area sampling fraction. Four coronal brain sections per animal were quantified at four different bregma levels (−1.06 mm, −1.46 mm, −2.10 mm and −2.46 mm), to give a total number of viable NeuN-positive cells. For quantification of rod microglia, a picture of a region of 1.1 mm^2^ in the CA1 and the somatosensory cortex regions was taken. The total cell number of rod microglia, defined as Iba-1 positive cells with highly polarized processes, was manually quantified and normalized to the area of the region of interest.

### RT-PCR analysis

Total RNA was extracted using the TRIzol reagent (Sigma Aldrich) following the manufacturer’s instructions. Using the RevertAid First Strand cDNA Synthesis Kit (Thermo Scientific, UK), 0.5 μg of the total RNA was transformed into cDNA. Quantitative RT-PCR was performed using the MESA BLUE SYBR® Assay (Eurogentec, UK) and primers listed in Supplementary Table S3. Results were calculated using the delta Ct method and represented as absolute values with arbitrary units.

### Measurement of galectin-3 in the cerebrospinal fluid (CSF) in mice

Cerebrospinal fluid samples were obtained from the *cisterna magna* using an insulin syringe in deeply anesthetised naïve, and at 2 and 24 h after head injury. Approximately 5 μl of CSF was obtained and fast frozen with dry ice and kept at −80 °C for further analysis. The amount of galectin-3 released present in the CSF from 3 μl samples was analyzed using the galectin-3 kit (Cat No. 12684, BG-Medicine, USA).

### Immunoprecipitation

The physical interaction between galectin-3 and TLR-4 was determined by immunoprecipitation analysis of brain tissue. The amount of 2.5 mg protein per sample was immunoprecipitated. We followed the method previously described^22^. Cells are lysed after treatment, homogenized with one pulse of 30 s sonication, and pelleted by centrifugation in buffer containing 20 mM Tris-HCl, pH 7.5, 140 mM NaCl, 1% Triton X-100, 2 mM EDTA, 1 μM p-amidinophenylmethanesulfonyl fluoride hydrochloride, 50 mM NaF, and 10% glycerol. The supernatants are pre-cleared with Dynabeads (Thermo Fisher Scientific) for 4 h at 4 °C. Later on, the cleared supernatants are incubated with 2.5 μg of anti-TLR4 antibody and normal rabbit IgG (R&D systems) as a negative control overnight. The next day, the lysates are incubated with Dynabeads for 4 h at 4 °C. The immunoprecipitate were washed four times with buffer containing 50 mM Tris-HCl, pH 7.5, 0.1% SDS, 1% NP-40, and 62.5 mM NaCl and subsequently dissolved in denaturing sample buffer. All cell extracts were processed for immunoblotting with a SDS-polyacrylamide gel electrophoresis as described in a 12% Acrylamide/Bis-acrylamide gel.

### Image capture and analysis

The quantification of microglia morphology was performed using a specially written script for the ImageJ analysis program (ImageJ 1.50d, National Institutes of Health, Bethesda, Maryland), and incorporating the ImageJ plugin “AnalyzeSkeleton”. AnalyzeSkeleton counts branch points (junctions) and branch length in cells following skeletonization. A 163 × 102 μm frame was placed over different areas of interest in the hippocampus. Twenty cells per treatment were analysed using the script, to quantify the number of secondary branches and single, triple and quadruple junctions.

### Quantification of the cortical tissue loss

The macroscopic images of the coronal brain sections (between bregma +0.5 to −4.04) from 21 days post traumatic brain injury stained with toluidine blue, were captured on a digital eyepiece camera via a dissecting microscope. Briefly, sections were air dried and rinsed shortly in distilled water. After that, the slides were submerged in a solution of 300 ml of 70% EtOH with 5 ml of 0.2 M acetic acid for 2 hours. Thereafter, slides were incubated in a filtered 0.1% toluidine blue solution for 10 minutes and rinsed 3 times with 1 minute distilled water washes. Slides were dehydrated through a series of 2 minutes washes with increasing concentration of ethanol (1x with 70% EtOH, 2x with 96% EtOH and 3x with 99% EtOH). After a further 3 × 5 minutes incubation in 100% xylene, the slides were mounted using DPX mounting media. The brain images were taken from 3 slides per animal, each slide containing 4–5 sections. Images were analysed using ImageJ software. The region of brain tissue loss was drawn around in reference to the contralateral side of the brain section, and the area was converted to mm^2^ based on measurements captured on a graticule. Statistical analysis was carried out using Student’s t-test from n = 4 animals per group.

### Statistical Analyses

The differences between control and experimental groups were evaluated with two sided-*t*-tests or one-way ANOVA with post-hoc Fisher’s least significant difference (LSD) or Tukey’s analysis (more than 2 groups) where appropriate, with *P* < 0.05 was considered statistically significant.

## Additional Information

**How to cite this article**: Yip, P. K. *et al*. Galectin-3 released in response to traumatic brain injury acts as an alarmin orchestrating brain immune response and promoting neurodegeneration. *Sci. Rep.* **7**, 41689; doi: 10.1038/srep41689 (2017).

**Publisher's note:** Springer Nature remains neutral with regard to jurisdictional claims in published maps and institutional affiliations.

## Supplementary Material

Supplementary Figures and Tables
