# Supplementary material for: Galectin-3 released in response to traumatic brain injury acts as an alarmin orchestrating brain immune response and promoting neurodegeneration
Source: Sci Rep. 2017 Jan 27;7:41689. doi: 10.1038/srep41689 (PMC5269662; doi:10.1038/srep41689)

**Galectin-3 released in response to traumatic brain injury acts as an alarmin orchestrating brain immune response and promoting neurodegeneration**

Ping Kei Yip, Alejandro Carrillo-Jimenez, Paul King, Anna Vilalta, Koji Nomura, Chi Cheng Chau, Alexander Michael Scott Egerton, Zhuo-Hao Liu, Ashray Jayaram Shetty, Jordi Lopez Tremoleda, Meirion Davies, Tomas Deierborg, John V. Priestley, Guy Charles Brown, Adina Teodora Michael-Titus, Jose Luis Venero and Miguel Angel Burguillos.


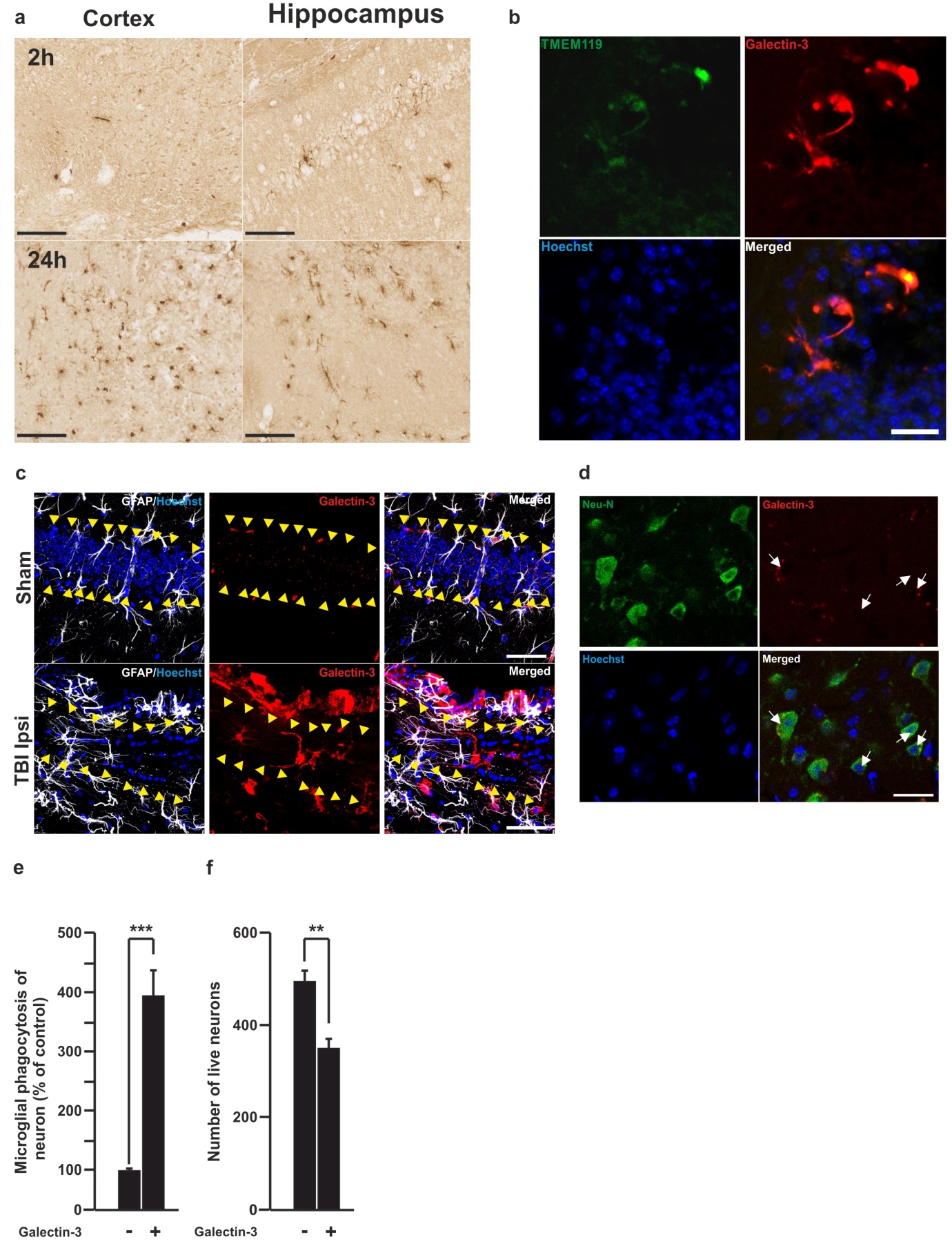


**Supplementary Figure S1: Galectin-3 expression in microglia and stressed neurons.**

Representative pictures of galectin-3 immunostaining in the ipsilateral cortex and hippocampus 2 and 24h after impact (a). Colocalization pictures of galectin-3 with TMEM119 in the dentate gyrus (b), GFAP in sham and TBI ipsilateral side in hippocampus (c) and NeuN in the Cortex (d) 24h after injury. Arrows in d show galectin-3 punctiform expression in NeuN positive cells in the cortex. Analysis of BV2 microglia cell phagocytic capacity after 2h incubation with neuronal debris in cells pre-incubated with recombinant galectin-3 (200nM) for 18 hours (e). Quantification of neuronal loss in rat primary mixed glial cultures treated with recombinant galectin-3 (1 μM) for 96 h (f). Mean ± s.d. n=3 for e and f. Data were analysed using Tukey’s post hoc test (e) and a two-sided Student’s t-test (f). **p<0.01 *** p<0.001. Scale bar for a is 100 μm, for b and d is 20 μm and for c is 50 μm. Triangles define the area occupied by neuronal cell bodies in the CA1 region. Hoechst dye was used to counterstain the nuclei.


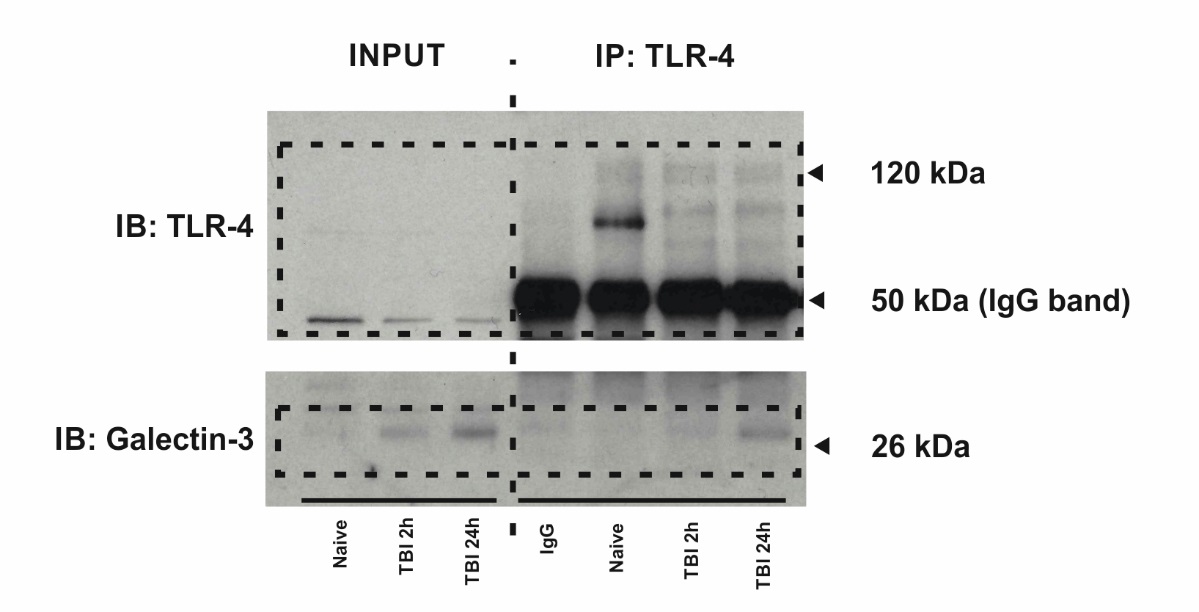


**Supplementary Figure S2: Full length blot from figure 2a.** Dashed line represents the cropped region shown in Figure 2a.


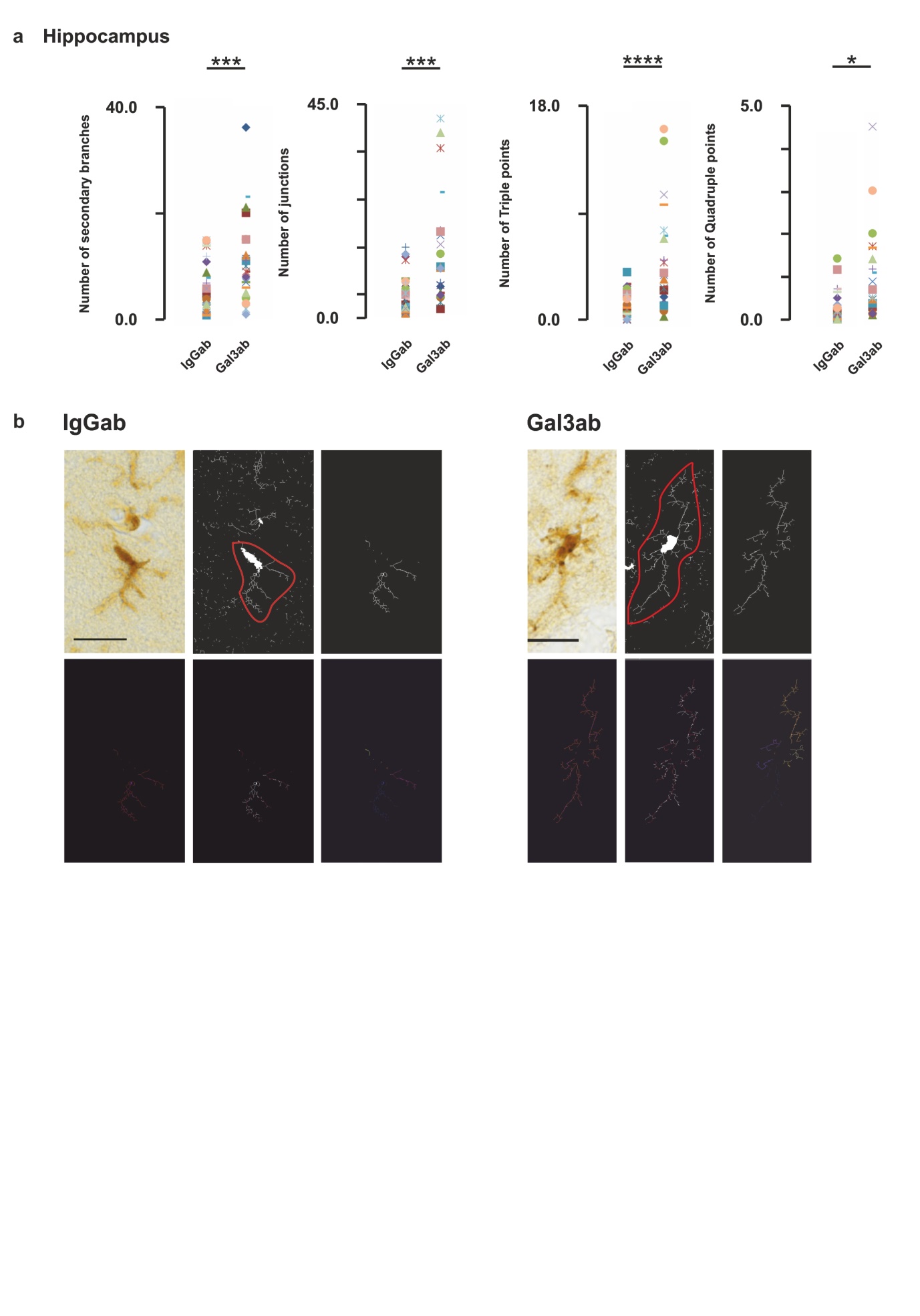


**Supplementary Figure S3: Analysis of the morphology of Iba-1 positive cells in hippocampus 24h after injury.**

Twenty cells per animal (n=3) per group were analysed using an ImageJ script incorporating the AnalyzeSkeleton plugin to assess whether there was any change in the reactivity (measured by morphology) between galectin-3 neutralizing antibody injected animals, compared to isotype control animals. Charts quantifying the number of secondary branches, junctions and triple and quadruple points in Iba-1 positive cells (a). Representative pictures of two Iba-1 positive cells in isotype control and galectin-3 antibodies that were analysed using the ImageJ script (b). Images show cells before, and after, skeletonization. *p<0.05 *** p<0.001 **** p<0.0001. Scale bar for b 12.5 μm.


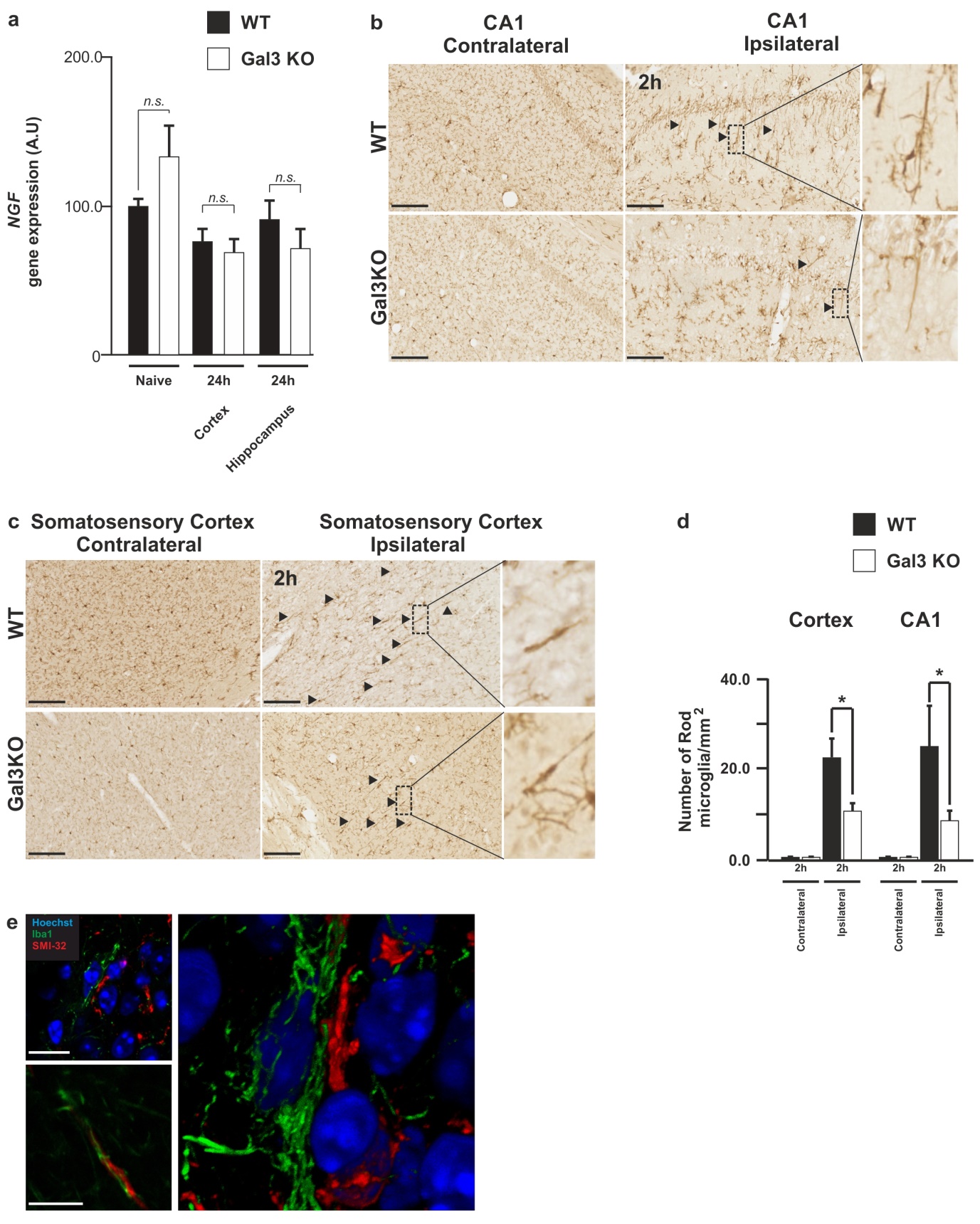


**Supplementary Figure S4: Distribution of rod microglia in the cortex and hippocampus 2h after injury in wild type and galectin-3 knockout mice.**

NGF gene expression in WT and galectin-3 knockout mice 24h after injury (a). Representative images of Iba-1 staining 2h after impact in the CA1 and the somatosensory cortex (b and c). Histogram showing quantification of rod microglia in those two areas at 2h after impact (d). Colocalization image of Iba-1 with SMI-32 in the CA1 region and the somatosensory cortex showing that rod microglia and neurons are in close proximity (e). Statistics and error bars: mean ± s.d. n=6 for A and n=4 for d. Data were analysed using a two-sided Student’s t-test. **p*<0.05. Scale bar in b and c is 100 μm and for e is 15 μm. Hoechst dye was used to counterstain the nuclei.


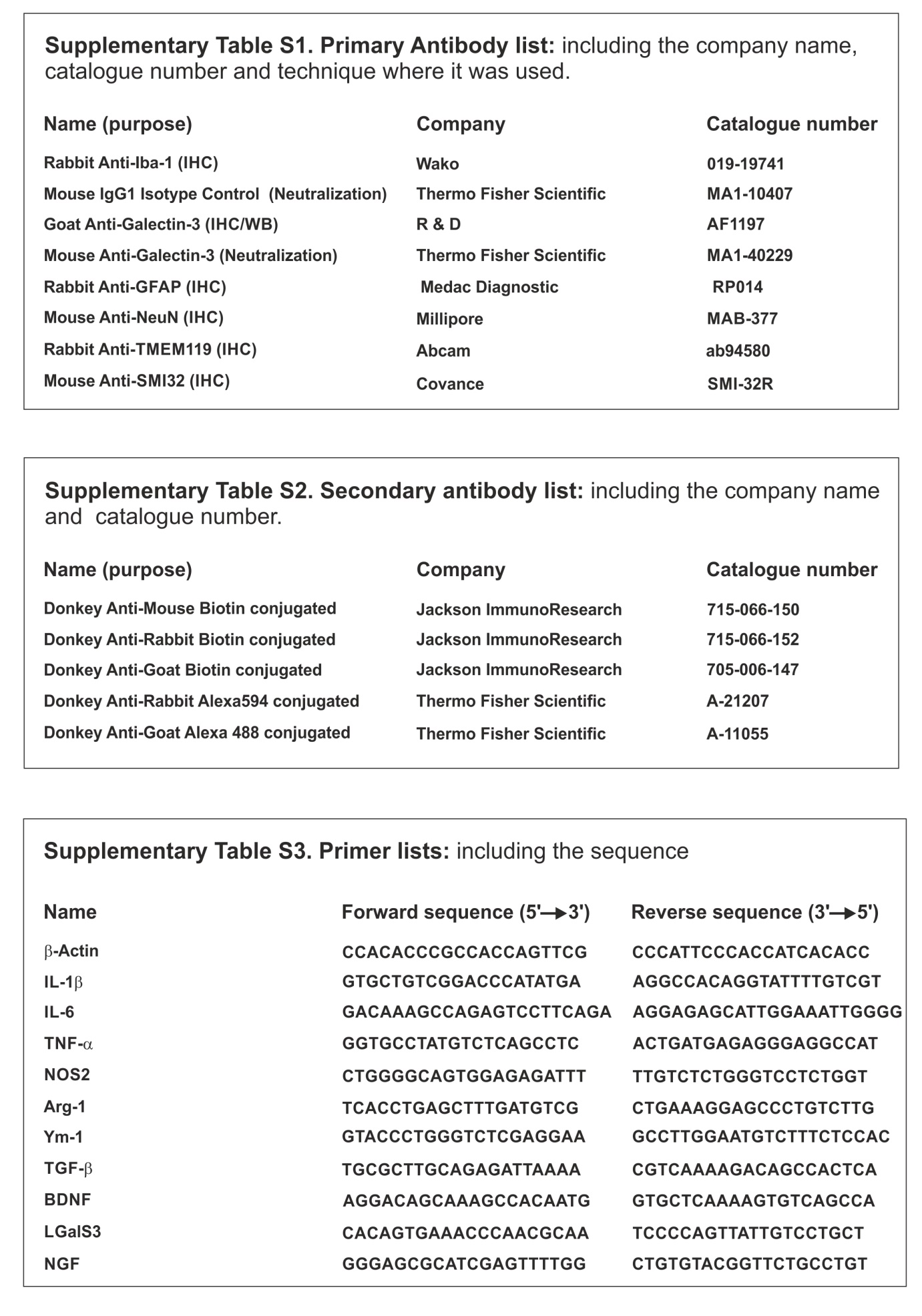

Supplement: Supplementary Figures and Tables [file srep41689-s1.doc]
